# Homœopathy and Rational Medicine

**Published:** 1901-01-12

**Authors:** 


### HOMŒOPATHY AND RATIONAL MEDICINE.

In an article entitled "A Century of Homœopathy" Dr. R. E. Dudgeon,[1] among other exercises, seeks to explain the fact that of late years there has been some arrest of the "flowing tide of homœopathy." Among other things he says that during the last fifty years there has been "such a *rapprochement* in the practices of the two schools that there is not that vast difference between them which was notorious in the first half of the century," the suggestion of course being that the methods of rational medicine have approximated to those of homœopathy—for how could homœopathists desert principles which, if true at all, are true for all time? Because ordinary physicians make use of certain drugs which homœopathists also employ he makes the extraordinary claim that we have "pillaged" their materia medica. Homœopathetic materia medica indeed! As if the fact of any substance having been used in homœopathy or any other pathy could stand in the way of its employment in ordinary rational medicine if it could be shown to do good. It is the confusion of thought among the public about what people choose to call "homœopathic remedies" that is at the root of much of their misunderstanding on the subject. Ignorant people think that aconite, nux vomica, &c., are "homœopathic remedies," and in some vague way belong to the homœopathists; and Dr. Dudgeon merely plays upon this ignorance when he says, "We find them (that is ordinary practitioners of medicine) now employing many medicines which were unblushingly taken from homœopathic sources, such as aconite, nux vomica, pulsatilla, bryonia, drosera, nitro-glycerine, and other strange drugs, and prescribing many of the old medicines on the indications given by homœopathic practitioners in doses of hitherto undreamt-of smallness. . . . Thus much of old-school practice is a sort of homœopathy."

But no one knows better than Dr. Dudgeon that the giving of small doses or the use of any particular sort of drug is not homœopathy. When we find Dr. Dudgeon speaking of the *rapprochement* of the "two schools," and of our pillaging the homœopathic materia medica, we must reiterate what we should have thought that everybody knew, namely, that practitioners of rational medicine are not restricted in any way in their choice of remedies, so only that they have reason to believe that they will do good to their patients. Whether the beneficial effect of a certain drug is discovered by a homœopathist, an old woman, or a naked savage is a matter of complete indifference to a practitioner of rational medicine; all he wants to be satisfied about is whether it really does good. That mesmerism is the basis of much arrant quackery does not prevent us from using suggestion; that osteopathy is sheer humbug, and that bone-setting has for ages been practised by the most ignorant, does not prevent us from getting all the good that is to be got out of massage, and "movement," and other mechanical methods; and the fact that we have no conception how light acts in the cure of lupus does not stand in the way of its introduction into our largest hospitals. All we wish to be convinced of is that these things do good. It is a matter of complete indifference to us whether the use of aconite in disease was originally discovered by chance, or by experiments on dogs, or by homœopathic "provings" on healthy people. None of these are reasons which influence us in using it, but merely the accumulated experience of its beneficial action in certain morbid states and of its power to produce certain effects which we desire for the relief of our patients.

Homœopathy is a particular system by which to discover remedies for certain conditions or symptoms, and more or less loosely attached to it are certain methods of dosage; and if the homœopathists can by "provings" discover any remedy that has not been known before for any condition, all well and good; let us give them every credit for it. But as soon as it is shown that it does good, not by "provings" in which we have no faith, but by experience, it is as much at the disposal of the rational physician as if it had been discovered in a laboratory or found in use in distant quarters of the globe. The range of homœopathy is strictly limited to the "provings" worked out according to the system; all outside this is a blank, often filled up by that other strange device, eclectic medicine. Rational medicine, however, embraces the whole field of experience, including even the homœopathic materia medica. But when the ordinary practitioner gives his dose of aconite or of bryonia, he does so, not because of the results of any number of "provings" on healthy persons, but because he believes, as the result of experience in regard to the sick, that it will do good, and in thus acting he in no way practises homœopathy. That in one sense, however, there has been a certain degree of *rapprochement* between the "two schools" we do not deny. The rank and file of the profession of medicine have nothing to do with experiments on monkeys, any more than the rank and file of the homœopathists have to do with "provings." Both alike trust mainly to the teaching of their leaders, and both alike constantly throw on one side such teaching when they find that in practice it does not do what it promised. Thus it happens that in the ordinary practice of their profession both alike are constantly learning from experience, and the homœopathist is constantly correcting the teachings of his principles by the teachings of his own observation of disease; in other words, he is constantly drifting towards rational medicine. It is an unconscious process no doubt, but directly a homœopathist deserts the teachings of his "provings," and appeals to his own experience in the choice of a drug, he deserts the principles of homœopathy and accepts those of rational medicine. What particular cupboard he takes his physic out of does not matter in the slightest. No man, unless he is a fool, refuses the lessons of experience, and thus there is some *rapprochement*. But it is on the side of the homœopathists; and this is a sore point.

[1] Monthly Homœopathic Rev.

